# Use and knowledge of Cactaceae in Northeastern Brazil

**DOI:** 10.1186/1746-4269-9-62

**Published:** 2013-08-28

**Authors:** Camilla Marques de Lucena, Reinaldo Farias Paiva de Lucena, Gabriela Maciel Costa, Thamires Kelly Nunes Carvalho, Gyslaynne Gomes da Silva Costa, Rômulo Romeu da Nóbrega Alves, Daniel Duarte Pereira, João Everthon da Silva Ribeiro, Carlos Antônio Belarmino Alves, Zelma Glebya Maciel Quirino, Ernane Nogueira Nunes

**Affiliations:** 1 Departamento de Fitotecnia e Ciências Ambientais. Setor de Ecologia e Biodiversidade, Universidade Federal da Paraíba. Centro de Ciências Agrárias. Laboratório de Etnoecologia, Areia Postal Code: 58.397-000, Brasil; 2 Departamento de Biologia, Universidade Estadual da Paraíba. Centro de Ciências Biológicas e da Saúde. Avenida das Baraúnas, 351. Campus Universitário. Bodocongó. Campina Grande, Paraíba Postal Code: 58.109-753, Brasil; 3 Departamento de Fitotecnia e Ciências Ambientais, Universidade Federal da Paraíba. Centro de Ciências Agrárias. Setor de Tecnologia Ambiental, Areia Postal Code: 58.397-000, Brasil; 4 Departamento de Geografia e História, Universidade Estadual da Paraíba. Centro de Humanidade. Rodovia PB 075, 2001. Zona Rural, Guarabira Postal Code: 58.200-000, Brasil; 5 Departamento de Engenharia e Meio Ambiente, Universidade Federal da Paraíba. Centro de Ciências Aplicadas e Educação. Tv. Manoel Gonçalves I, Rio Tinto Postal Code: 58.297-970, Brasil

**Keywords:** Cacti, Ethnobotany, Use value, Semi-arid

## Abstract

**Background:**

This study aimed to record the use, and knowledge that residents from São Francisco community (Paraiba, Brazil) have regarding the Cactaceae.

**Methods:**

Semi-structured interviews were carried out with 118 informants; 50 men and 68 women. The cacti cited in this study were organised into use categories and use values were calculated. Differences in the values applied to species and use categories by men and women were compared via a G test (Williams).

**Results:**

The nine species identified were: *Cereus jamacaru* DC., *Melocactus bahiensis* (Brtitton & Rose) Luetzelb., *Nopalea cochenillifera* (L.) Salm-Dyck., *Opuntia ficus indica* (L.) Mill, *Opuntia stricta* (Haw.) Haw., *Pilosocereus gounellei* (F.A.C. Weber) Byles & Rowley, *Pilosocereus pachycladus* F. Ritter, *Tacinga inamoena* (K. Schum) N.P. Taylor & Stuppy, *Tacinga palmadora* (Britton & Rose) N.P. Taylor & Stuppy. In total, 1,129 use citations were recorded, divided into 11 categories. The use value categories with the highest scores were forage (0.42), food (0.30) and construction (building) (0.25). *P. pachycladus* showed the greatest use value, versatility and number of plant parts used.

**Conclusion:**

The survey showed that the Cactaceae is extremely important for several uses and categories attributed to different species. Apart from contributing to the ethnobotanical knowledge of the Cactaceae, another important focus of this study was to reinforce the necessity for further studies that record the traditional knowledge about this plant family, which has been lost in younger generations.

## Background

Despite the most widespread image of the Caatinga as belonging to a poor region, it is one of the richest regions in Brazil, regarding plant species number when compared to other ecosystems. Additionally, the region hosts a large number of endemic vegetable species, especially among the Cactaceae family. Due to this endemic feature and the high degree of environmental degradation due to anthropogenic activities, the Environment Administration Government has recognized the Caatinga as an area that requires conservationist actions [1]. A list of the most vulnerable species (Normative Instruction N° 06/ 2008) includes 28 cacti, and especially notable in this list is the genus *Melocactus*, with five endangered species in Bahia state [2].

The prominence of cacti in the Caatinga is due to their morphological and physiological adaptations to low rainfall, a limiting factor for living in a semi-arid region. Among these adaptations are the juicy stem, which stores water during the drought season, short stature, and leaves that have evolved into spines, which avoid water loss by evaporation [3]. Cacti are characterized as xerophytic plants, juicy, often thorny, with mucilaginous and watery tissue, the stomata are protected to diminish the superficial perspiration and roots absorb the night dew [4]. Cacti have a photosynthetically active stem, of variable colour, shape and size, forming cladodes, which can be plain, cylindrical, columnar or globular and are usually coated with thorns. Regarding columnar cladodes, these have a central part, with a radial structure which forms from three to many angles [5-7].

The Cactaceae family is divided into four subfamilies: the Maihuenioideae, Pereskioideae, Cactoideae and Opuntioideae [8] that are spread throughout the American continent, in tropical and temperate regions, with the highest diversity of species occurring in Mexico. In Brazil, there are 40 genera and 200 species [9], of which 24 genera and 88 species are present in the Northeastern region [10]. These Northeastern species are differentiated from those of the South and Southeastern states, with Bahia state being the dispersion centre [11]. According to Barroso *et al.*[12], the difference between these two Brazilian groups is due the fact that the cacti from the northeastern region are similar to those species from North America, whereas those from the southern region maintain the characteristics of the South American species. Both groups are found in Brazilian semi-arid areas, such as *Cereus jamacaru* DC. (mandacaru), *Pilosocereus gounellei* (F.A.C. Weber) Byles & G.D.Rowley (xiquexique), *Melocactus bahiensis* (Britton & Rose) Werderm. (coroa de frade) and *Pilosocereus pachycladus* F. Ritter (facheiro) [13], as well as exotic species; e.g. *Opuntia ficus indica* (L.) Mill (palma forrageira) and *Nopalea cochenilifera* (L.) Salm-Dyck (palma doce).

According to Andrade *et al.*[14,15], apart from agriculturists who live in the northeastern semi-arid region and have a vast knowledge about the Cactaceae, few studies with an ethnobotanical focus have been performed to record the knowledge and diversity of uses applied to the species by the traditional population. In this sense, ethnobotany might considerably contribute to the recording of knowledge and use that agriculturists have about cacti and at the same time, provide information for the development and application of the sustainable handling of these plants. In other regions of the world, ethnobotanical studies on cacti are being initiated, e.g. in Mexico [16-22], Cuba [23], Colombia [24] and the United States [25], to analyse information concerning varied aspects of use, management and species domestication.

In Mexico, many species have been subjected to domestication due to the intensive use of fruit [18]. In Cuba, a medicinal use has been noted for several species [23] and in Colombia, one important use is that of fruit as human food [26] and also, in the United States, some species have a cultural value and are also used as childrens’ toys [25].

One of the potential uses of cacti is as fodder species for cattle, sheep and goats during the drought season, together with native grass, with the aim of fattening the flock and increasing milk production [4,26]. Additionally, some industrialised products such as shampoo and soaps are produced mainly from the species *O. ficus indica* (forage cactus). Species such as *P. gounellei* (xiquexique), *P. pachycladus* (facheiro) and *M. bahiensis* (coroa de frade) are used to make biscuits, coconut candy, pudding, cakes and candies, and thereby provide a source of income for rural communities [14,27]. Cactus use in local medicine is also prevalent in rural communities in the treatment of illnesses such as infections, flu, urethra problems and worms [14] and use is also observed in home construction in many communities, as well as in the production of laths of *P. pachycladus* used in the building of roofs of houses [26,28].

Further ethnobotanical studies are necessary, following the current trend of quantitative ethnobotany and economic botany, seeking to test hypotheses that relate to the knowledge and use of cacti, such as the hypotheses of ecological apparency [29,30], and optimal foraging [31,32]. What has determined the use and selection of these species? The selection is the choice of the species most preferred by people. Are there differences in knowledge and use by men and women? Are all the potential uses of cacti known, for both food and medicinal contexts? Have the species that have been used for medical purposes proven medicinal properties? Why are cacti so appreciated as ornamental plants? Has the spread of exotic cacti caused any ecological or other impact upon native species? All these questions might inform and direct future ethnobotanical research of this plant group. However, it was not used in this manuscript.

In view of the above and the utility shown by cacti, the present study aimed to record the knowledge and use by inhabitants of the São Francisco community in Cabaceiras city (Paraiba, Brazil), regarding the cacti found in the region.

## Materials and methods

### Study area

**Figure 1:**
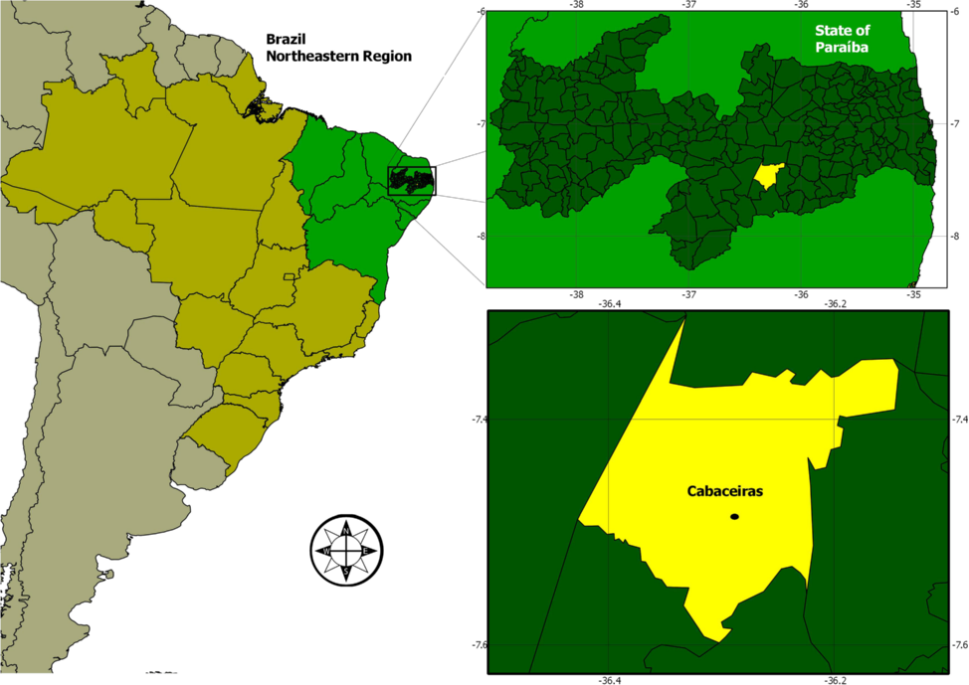
Map of study area in Cabaceiras city, Brazil.

The study was performed in the São Francisco rural community, in Cabaceiras city, Paraiba state (Northeastern Brazil) (Figure 1). This community is at approximately 400 m altitude and is located in the Meso-region from Borborema and micro-region from Eastern Cariri, 7°29’20”S and 36°17’14”W [33], situated 66.7 km from Campina Grande city (a regional hub) and 199 km from João Pessoa (the state capital). It has 5,035 inhabitants, 2,217 being in the urban zone and 2,818 in the rural zone, in an area of 452,920 km^2^[34]. The climate is BSh (hot semi-arid), with a mean annual temperature exceeding 20°C and the lowest rainfall index in Brazil, with less than 300 mm of rain during the entire year [34]. The vegetation of the region is of the Caatinga hiperxerophilous type, being mostly characterised by the family Cactaceae and Bromeliaceae.

### São Francisco community

The São Francisco community is divided into five localities called Caruatá de Dentro, Jerimum, Alto Fechado, Rio Direito and Malhada Comprida [35]. It has a basic education state school and a Catholic chapel located at Caruatá de Dentro, called São Francisco chapel. One of the main local economy sources is farming, including raising goats and maize and bean cultivation.

### Collection of ethnobotanical data

**S**emi-structured interviews were performed with 118 informants (householders), in 72 homes, with both men (n = 50) and women (n = 68), in separate interviews [36]. The difference between the number of men and women occurred because of differing marital status’, due to the presence of widows or widowers, single or divorced respondents. Each householder was considered as an informant. Before the start of each interview, each informant was explained the goal of this study and was requested to sign a Term of Free and Clarified Assent, demanded by the Health National Council through the Ethics and Research Committee (Resolution 196/96 of the CNS/MS). The study was developed and approved by the Committee of Ethics in Research with Human Beings (CEP) of the Lauro Wanderley Hospital from the Federal University from Paraiba, registered in protocol CEP/HULW n° 297/11.

The community contained 88 habitable residences, which were all visited. Only in one residence did the residents decline to participate. For 15 other residences, the inhabitants could not be found, after being visited three times on average in an attempt to perform the interviews.

The form used to obtain the data involved questions referring to the knowledge of the informants about the use of the Cactaceae species found in the region: Which cacti do you know? Are any cacti used for building? Are any cacti used medicinally? Are there any cacti used for animal feeding? Who taught you? Do you teach anyone about cacti?

The cacti mentioned were organised into use categories adapted from the literature [14,37-40]. In each one of these categories (food, fuel, building, fodder plant, medicinal, ornamental, technology, veterinarian, shade, religious magic and personal hygiene), sub-categories were included, to indicate uses that were defined precisely and objectively, according to the course of the interviews. Collected field specimens were incorporated into the Herbarium Jaime Coêlho de Moraes (EAN) of the Federal University of Paraiba, at the Agrarian Sciences Center.

### Data analysis

For each species and use category, the use value was calulated according to the formulae VU = ∑ Ui/n and VUc = ∑ VUs/nc, described by Rossato *et al*. [41], where: Ui = number of uses mentioned for each informant, n = total number of informants, VUc = use value of each species in the category, VUs = use value of each species in the family, and nc = number of species in the category. When the use value of the species for men and women was calculated, the calculation was performed separately i.e., it was considered as the number of uses cited by all men, divided by the total number of men, the same procedure being adopted for women.

Differences in the values attributed to the species and the use categories for men and women were compared using the G test (Williams) [42].

## Results

### Ethnobotanical inventory of the cactaceae

**Figure 2:**
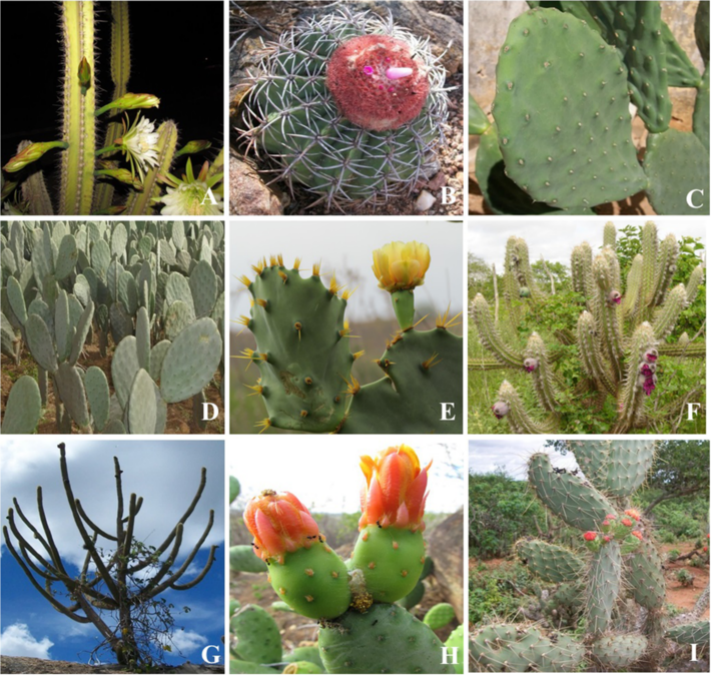
**Cactus species cited by community residents of São Francisco in Cabaceiras city, Brazil. A**. *Cereus jamacaru* DC.; **B**. *Melocactus bahiensis* (Britton & Rose) Luetzelb.; **C**. *Nopalea cochenillifera* (L.) Salm-Dyck.; **D**. *Opuntia. ficus indica* (L.) Mill.; **E**. *Opuntia stricta* (Haw.) Haw.; **F**. *Pilosocereus gounellei* (F.A.C.Weber) Byles & Rowley; **G**. *Pilosocereus pachycladus* F. Ritter; **H**. *Tacinga inamoena* (K. Schum.) N.P. Taylor & Stuppy; **I**. *Tacinga palmadora* (Britton & Rose) N.P. Taylor & Stuppy.

Nine species, belonging to six genera were recorded: *Cereus jamacaru* DC. (mandacaru), *Melocactus bahiensis* (Britton & Rose) Luetzelb. (coroa de frade), *Nopalea cochenillifera* L.Salm-Dyck. (palma doce) *Opuntia. ficus indica* (L.) Mill (palma forrageira), *Opuntia stricta* (Haw.) Haw. (palma de espinho), *Pilosocereus gounellei* (F.A.C. Weber) Byles & Rowley (xiquexique), *Pilosocereus pachycladus* F. Ritter (facheiro), *Tacinga inamoena* (K. Schum) N.P. Taylor & Stuppy (cumbeba), and *Tacinga palmadora* (Britton & Rose) N.P. Taylor & Stuppy (palmatória) (Figure 2). The number of different use citations was 1,129; 593 by women and 536 by men, which were organised into eleven use categories according to their utility.

**Figure 3:**
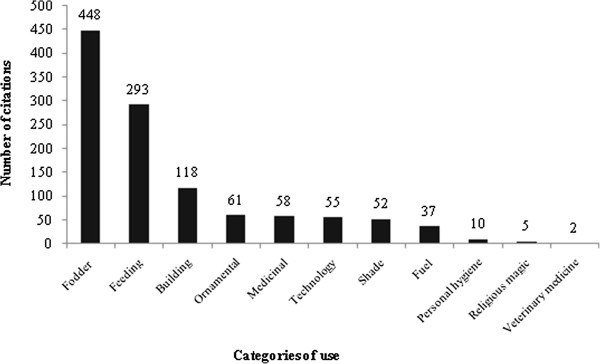
Number of citations of use by utilitarian category of cactus cited in the community of São Francisco in Cabaceiras city, Paraíba (Northeastern Brazil).

The most-mentioned species were *P. pachycladus* (273 citations), *P. gounellei* (227), *O. ficus indica* (210) and *C. jamacaru* (175), followed by *M. bahiensis* (126 citations), *T. palmadora* (84), *T. inamoena* (26), *O. stricta* (6) and *N. cochilinifera* (2). The use categories highlighted were: fodder, with 448 citations, representing 40% of the total, food with 293, building with 118, and ornamental with 61 (Figure 3). The use values of the above species, were *P. pachycladus* (2.31), *P. gounellei* (1,92), *O. ficus indica* (1.77), followed by *C. jamacaru* (1.48), *Melocactus* sp. (1.06), *T. palmadora* (0.71), *T. inamoena* (0.22), *O. stricta* (0.05) and *N. cochenillifera* (0.01).

Forage was the category with the highest use value (0.42), followed by food (0.30), building (0.25), ornamental (0.07), medicinal (0.10), technology (0.19), shade (0.10), fuel (0.15), religious magic (0.02), personal hygiene (0.03) and veterinarian (0.008).

Regarding versatility, *P. pachycladus* and *C. jamacaru* fitted into eight of the eleven use categories, *P. gounellei* into seven, *O. ficus indica* into six, *M. bahiensis* into five, *O. stricta* into four, *T. palmadora* into three, *N. cochenillifera* into two and *T. inamoena* into one.

Analysis of the use of the different plants parts of individual species, showed that *P. pachycladus* and *C. jamacaru* had five useful parts, *O. ficus indica* and *P. gounellei* four, *M. bahiensis* and *T. palmadora* three, *O. stricta*, *N. cochenillifera* and *T. inamoena* two. The most-used part was the “entire individual” present in 474 citations and this use represents the cutting of the whole plant for subsequent burning and feeding to animals. After that, the wood received 178 citations, fruit 152, marrow (torch) 108, pulp (marrow) and rackets (cladode) 97, root with 11 and the gum (parenchyma) with six citations.

Amongst the seven native species recorded, *P. pachycladus* had the highest number of citations for building use.

Amongst the exotic species recorded, *O. ficus indica* was most used as forage, its cut cladodes being used for animal feed.

### Use of the cactaceae

In the studied community, various ways of using of cacti found in the region were recorded, involving uses such as for timber or non-timber. All informations of use are presented in Table 1. Below are described some usage information for each species.

**Table 1:** Categories of use of cactus species cited by community residents of São Francisco in Cabaceiras city, Brazil

**Species**	**Vernacular name**	**Voucher ufpb**	**Categories of use**	**Part used**	**Mode of use**	**Utilization**
*Cereus jamacaru* DC.	Mandacaru	17619	Feeding	Fruit	*In natura*	
			Fuel	Wood	Firewood	
			Building	Wood	Laths, boards, doors and window	
			Fodder plant	Entire plant	Burnt or cut	
			Medicinal	Marrow	Syrups	Cough, column, wound, furuncle, urinary infection, inflammation, kidney inflammation, rheumatism e urethra
				Root	Tea	
			Ornamental	Entire plant	Planted in the gardens and yards	
			Shade	Entire plant		
			Technology	Wood	Handle of tool	“Chibanca”
*Melocactus bahiensis* (Britton & Rose) Luetzelb.	Coroa de frade	17572	Feeding	Fruit	*In natura*	
			Fodder plant	Entire plant	Burnt or cut	
			Religious magic Medicinal	Entire plant	Put into jars at home	Evil eye
				Marrow (aquifer parenchyma)	Syrups	Amoeba, catarrh, whooping cough, cough e worm
			Ornamental	Entire plant	Planted in the gardens and yards	
*Nopalea cochenillifera* (L.) Salm-Dyck	Palma doce	19765	Feeding	Fruit	*In natura*	
			Fodder plant	Leaf (rackets)	Cut	
*Opuntia ficus indica* (L.) Mill.	Palma forrageira	19769	Feeding	Fruit	*In natura*	
				Marrow	Cooked or raw	Cake, cooked with beans, with rice or with meat, candy, stew, jelly, risotto, salads, soup and juice
			Fodder plant	Leaf (rackets)	Cut	
			Personal hygiene	Marrow	Hair conditioner, soap, bath soap, shampoo	
			Religious magic	Entire plant	Placing the leaf (racket) in the bedroom of a sick person (asthma)	Asthma. Whooping cough and cough
			Medicinal	Marrow	Syrups	
			Shade	Entire plant		
*Opuntia stricta* (Haw.) Haw.	Palma de espinho	19764	Feeding	Fruit	*In natura*	
			Building	Entire plant	Hedges (fences)	
			Fodder plant	Entire plant	Burnt	
			Ornamental	Entire plant	Planted in the gardens and yards	
*Pilosocereus gounellei* (F.A.C. Weber) Byles & Rowley	Xiquexique	17629	Feeding	Fruit	*In natura*	Coconut candy, couscous, candy and flour
				Marrow	Baked or cooked	
			Building	Entire plant	Hedge	
			Fodder plant	Entire plant	Burnt	Remove the thorns from skin
			Personal hygiene	Marrow	Shampoo	
			Medicinal	Dribble	Through the affected area	Assist in the process of “not choke”
			Ornamental	Entire plant	Planted in the gardens and yards	
			Veterinary medicine	Dribble	Insert dribble in the animal throat	
*Pilosocereus pachycladus* F. Ritter	Facheiro	19616	Feeding	Fruit	*In natura*	Candy and coconut candy
				Marrow	Baked or cooked	
			Fuel	Wood	Firewood	
			Building	Wood	Fences, “door guard”, door, gates, laths, boards	
			Fodder plant	Entire plant	Burnt or cut	
			Medicinal	Entire plant	Cooked	Anemia
			Ornamental	Entire plant	Planted in the gardens and yards	
			Shade	Entire plant		
			Technology	Wood	Handle of tool	Hoe, axes, hammers and pickaxe
					Roll of mass	
					Canoe	
					Oar	
					Oil lamp (burning wood)	Illuminate the nigth
*Tacinga inamoena* N.P. Taylor & Stuppy	Cumbeba	19766	Fodder plant	Entire plant	Burnt or cut	
				Fruit	*In natura*	Rhea
*Tacinga palmadora* (Britton & Rose) N.P. Taylor & Stuppy	Palmatória	17573	Feeding	Fruit	*In natura*	
			Fodder plant	Entire plant	Burnt	
				Leaf (rackets)	Cut	
			Ornamental	Entire plant	Planted in the gardens and yards	

*Cereus jamacaru* is used for cooked human food or as an ingredient in candies and its fruit is consumed fresh. Its wood is used in construction, for making doors, windows, boards and laths. As part of its medicinal use, it can be used as a tea, being prepared from the root and used to treat illnesses such as rheumatism, wounds, boils, urinary infections and kidney inflammation.

*Pilosocereus pachycladus* has its core (marrow) cooked or baked and is used as human food, for making candy including coconut candy, or its raw fruit is consumed. A further use for its wood is as firewood to light domestic stoves, and in the manufacture of laths, “door guards”, fences, doors, gates and boards.

*Opuntia ficus indica* is used for several purposes, the main one being for forage. It is also used in local cuisine, for cakes, candies, juices, jellies, soups, salads, stews, risottos or cooked with rice, beans and meat. The fruit is considered one of the best among all the Cactaceae, being also commercialized as “figo da Índia”.

In the case of *P. gounellei*, its core (marrow) can be consumed either baked or cooked, becoming candy or flour for couscous preparation. The same plant part can be made into shampoo, or its raw fruit consumed. For fodder use, the entire plant is burnt and served to animals; alternatively, it can be used in gardens as an ornamental.

The types of uses recorded for *Melocactus sp.* include as fresh fruit for human food, the whole plant is burnt or cut for fodder or used as an ornamental or for religious magic uses (evil eye and envy) and the kernel (aquifer parenchyma) is used in the preparation of syrup used to combat whooping cough, phlegm and amoeba.

The fruit of *T. palmadora* is used as a human food; it can also be used solely as an ornamental, or also burnt and/or cut to feed animals.

*Opuntia stricta* is used in the composition of hedges (fences), and its fruit serves as human food. It is also burnt and used to feed animals, and used as an ornamental in gardens and yards.

The fruit of *N. cochenillifera* serves as a food for humans, and as fodder (rackets).

*Tacinga inamoena* is only used as fodder, being burned or cut down and offered to animals, or fed as a fresh fruit.

### Evaluation of species by men and women

A comparison of the information provided by men and women showed that the most significant categories of fodder, feeding and building were the same for both groups. When analyzed using the G test, the number of citations attributed to the species and the categories of use for men and women (Test G (P) = 0.1343), didn’t differ significantly, indicating that men and women tended to categorize the species in a similar way.

## Discussion

### Use of the cactaceae

The diversity of species and their uses registered in the present study were also noted in other studies, both in Brazil [14,15,43-45] and in other countries, such as Cuba [23]. Amongst these uses were those involving fodder and human food. However, the Cuban study diverged regarding the main recognized categories in the present study. In the Cuban community, the most obvious categories were medicinal and ornamental, with food being in third position, which unified uses for humans and animals [23]. However, a more detailed analysis is necessary in this context, because the category food is relevant in Cabaceiras as the use of cacti for food plays an important role in the cultural mentality of people from semi-arid areas, even if using cacti for food is becoming less important, due to the fact that the local population is undergoing a social economic change as a result of governmental assistance, e.g., scholarships and family grants [35], suggesting an improvement in family income.

The use of cacti as a fodder plant is common, mainly during times of water shortage, when other forage species, such as those of the Poaceae family, are scarce, and cacti assume the main role in feeding the local flocks. As well as in the present study, the use of these plants as fodder plants mainly during drought periods, has also been noted by other researchers in Brazil [14,26,35,36,43,44,46]. Among fodder species, *O. ficus indica*, is predominant as a source of income, which stimulates its cultivation by agriculturists for their own use and/or for commercialization.

Medicinal and therapeutic uses were also well-recorded in the São Francisco community. The medical potential shown by cacti is mentioned in other studies, which demonstrate their various uses in the treatment of human illnesses [15,47-52]. In terms of veterinarian use, only a single species, *P. gounellei* was mentioned in this study. Andrade [53] reports that the same species is used medicinally by the “Sertanejos Baianos” people, being their “baba” mixed with castrated sheep mutton to remove stick tip off the skin.

Regarding the use of the cacti in domestic or rural constructions, the present study found considerable diverse uses, such as in the construction of roofs (laths), or to make gates. Such lumber uses are also noted in other semi-arid regions, as in Ceará [43], Bahia [14] and Paraiba [26]. A further lumber category that was registered in Cabaceiras was technology, for example, in the manufacture of handles of tools. However this use is little recognized in rural communities from semi-arid regions. Other uses recorded included the use of roots of *P. pachycladus* to make wooden spoons [43] and the hair (wooly trichomes) of *M. bahiensis* used in the wadding of the “cangalhas” (a type of saddle) for donkeys [44]. The prominence of certain species might result from their high use value, as the case of *P. pachycladus*, which was one of the most-cited species with great potential use by informants. Other researchers recorded uses for this species, thus confirming the findings in Cabaceiras, by Albuquerque *et al*. [36], who showed the culinary use (candy) in the Agreste from Pernambuco; Braga [43] highlights technological uses (wooden spoons made with the root), construction (boards), fodder plants and human food (fruit); and Gomes [46] describes forage uses.

*P. gounellei* demonstrated similar nutritional uses to those of *P. pachycladus*, in the daily life of Sertanejos Baianos who mainly consume its fruit [14]. Apart from this utilitarian potential, in Cabaceiras, it was also recorded as being useful for building “hedges”, that could also then be surrounded by barbed wire. In Cuba, other cactus species with a similar use were recorded, such as *Cereus hexagonous*, *O. ficus indica* and *N. cochenillifera*, being associated with wooden props and wire [23], underlining the potential of cacti in this type of rural construction. Other studies have also listed uses for *P. gounellei*, such as for fodder [43,44,46,52], ornamental [44,52] or medicinal use [15,52], findings that confirm the uses in the community studied here.

*O. ficus indica*, as well as being a fodder plant, also showed a great variety of nutritional uses, being used to make cakes, candies, juices, salads, soups, jellies, or being cooked with beans, meat and rice, as well as use of the fruit itself. This alimentary potential also was noted by Andrade *et al*. [14] in Bahia. Other interesting aspects of certain cacti are their use as bio-indicators of natural phenomena, such as the rainy season. If specific questions on such phenomena had been asked, other responses might have been elicited, however, only one informant cited the use of cacti as bio-indicators, as can be seen from this citation:

*“When the facheiro blooms with a yellow flower it is a signal that is going to rain”* (A.R.F. 29 years old).

The use of cacti as indicators has been recorded in other studies for *C. jamacaru* in Pernambuco [36], and in Paraiba for *P. gounellei*, *O. ficus indica* and for *C. jamacaru* in Sumé city [54] and Soledade [55].

### Evaluation of species by men and women

On average, men attributed greater use values and use categories to species, than women, even though the attributed values strongly correlated. This might be explained by the fact that men are more used to handling cacti, as they mainly cite uses related to animal feeding (fodder plants) than any other purpose, as found in the present study. Women were more familiar with uses for nutrition and personal hygiene, e.g. the use of *O. ficus indica* to make shampoo and bath soap. In semi-arid regions, there is a division of labour, with men being responsible for the cultivation of food and women for preparing it, as reported by Cavalcanti Filho [35]. This is shown by the following citations from informants:

*“My wife already made cooked palma forrageira; she already used shampoo, bath soap and cream”* (M.A.F., 45 years old).

“The stem of the facheiro serves as a board if it is thick, to make a boat and oars” (M.S., 45 years old).

When analysing this division of labour and knowledge between men and women, taking into consideration all locally-found vegetable species, the literature has shown that there is a tendency for men to be more familiar with lumber uses and the women with non-lumber uses [56-59]. This was also observed in Cabaceiras, where men mentioned many lumber uses for cacti, such as building roofs (laths) and for tool handles (e.g. hoes), and women mentioned many non-lumber uses, such as those in disease treatment. However, Lucena *et al*. [40], emphasises that the dynamics of knowledge and use of vegetable resources between men and women might vary from region to region and either be similar or distinct. Matavele and Habib [60] found a similar knowledge for men and women, mainly regarding the use of medicinal plants. This same situation was found in the present study, since men and women attributed the same values of use to the medicinal category. Figueredo *et al*. [61] showed that women have a distinct knowledge from that of men, principally regarding the use of medicinal plants. In the general context of the São Francisco community, it is noted that maintenance of the knowledge concerning the Cactaceae is very important for both men and women.

## Conclusions

The population from the São Francisco community demonstrated that the Cactaceae is extremely important due the diverse uses and categories attributed to different species. Exotic species as *Opuntia ficus indica* are increasingly being used by agriculturists as a fodder plant, as a new source of income within the community, and enabling the conservation of native species, such as *Pilosocereus gounellei* and *Pilosocereus pachycladus*.

The very high correlation of the use value between men and women demonstrates an equal knowledge for both sexes. In view of the demonstrated importance of cacti in this semi-arid region and of the richness of information acquired in the present study, it is suggested that similar studies should be performed to monitor knowledge within other communities and to allow a comparison with this study, as well as to consider the increased use and handling of cacti possibly via plantations, or through asexual propagation that would recognize the importance of these species in drought periods.

## Competing interests

The authors declare that they have no competing interets.

## Authors’ contributions

All authors read and approved the final manuscript.
